# Evaluation of the Integrative Weaning Index for Predicting the Outcome of Spontaneous Breathing Trial in Patients with Cirrhosis on Mechanical Ventilation: A Pilot Study

**DOI:** 10.5152/TJAR.2021.1057

**Published:** 2022-04-01

**Authors:** Shailesh Sahu, Vandana Saluja, Anamika Sharma, Lalita Gouri Mitra, Guresh Kumar, Rakhi Maiwall, Shiv Kumar Sarin

**Affiliations:** 1 Department of Anaesthesia and Critical Care, Institute of Liver and Biliary Sciences, New Delhi, India; 2 Department of Anaesthesia and Critical Care, Rajiv Gandhi Superspeciality Hospital, New Delhi, India; 3 Department of Hepatology, Institute of Liver and Biliary Sciences, New Delhi, India; 4 Department of Biostatistics, Institute of Liver and Biliary Sciences, New Delhi, India

**Keywords:** Cirrhosis, compliance, integrative weaning index, mechanical ventilation, rapid, shallow breathing index

## Abstract

**Objective:**

Studies on mechanical ventilation in patients with cirrhosis have focused mainly on survival, as the disease is considered to carry a poor prognosis. The process of weaning in these patients has never been studied. With improving survival, it would be ideal to study the weaning indices that could add experience in clinical management. The integrative weaning index is known to predict weaning failure, even in those who tolerate the spontaneous breathing trial. However, it has been evaluated mainly in patients with chronic obstructive pulmonary disease. Our aim is to study the integrative weaning index in predicting the outcome of the spontaneous breathing trial in patients with cirrhosis undergoing mechanical ventilation.

**Methods:**

Adult cirrhotic patients requiring mechanical ventilation for the first time were enrolled. Twenty-seven patients were found eligible for weaning. After the decision to wean, the patients were put on pressure support mode of ventilator followed by spontaneous breathing trial using T-piece for 2 hours.

**Results:**

The study population was divided into two groups: successful spontaneous breathing trial group (Group S) and unsuccessful spontaneous breathing trial group (Group U) based on the outcome of the breathing trial. The mean respiratory rate was significantly lower in Group S as compared to Group U. The compliance of the respiratory system and integrative weaning index were found to be significantly higher in Group S. An integrative weaning index of 28 mL/cmH_2_O breaths/min/L was found to be a good predictor of weaning success.

**Conclusions:**

Our results demonstrate that weaning from mechanical ventilation is possible in critically ill patients with cirrhosis. An integrative weaning index of 28 mL/cmH_2_O breaths/min/L could be a successful predictor of weaning from mechanical ventilation.

**Main Points**

- Weaning from mechanical ventilation is possible in critically ill cirrhosis.
- Threshold values of static compliance, rapid shallow breathing index, and IWI are different than the general population due to the peculiarities of the disease.
- Integrative weaning index (IWI) of 28 mL/cmH_2_O breaths/min/L is the most robust among all to predict weaning success.

## Introduction

Weaning covers the entire process of liberating the patient from mechanical ventilation and endotracheal tube.^1^ The prevalence of weaning in critically ill cirrhosis patients has been reported to be between 10% and 34%.^2^ Potentially modifiable factors like tense ascites, hydrothorax, encephalopathy, pulmonary infections, and volume overload can be treated to facilitate weaning in patients with cirrhosis. Some non-modifiable factors like sarcopenia, alpha^−1^ anti-trypsin deficiency, hepato-pulmonary syndrome, and portopulmonary hypertension may contribute to difficulties in weaning.^3,4^ The outcome of mechanical ventilation in cirrhosis was earlier considered to carry an extremely poor prognosis. Mortality was reported to be 83% within 72 hours of being placed on the mechanical ventilator in the 1990s.^5^ After decades of managing critical patients with cirrhosis, coupled with early admission to the intensive care unit (ICU), survival to ICU discharge has improved to 55%, with a 90-day survival of 48%.^6^ Studies have shown that respiratory failure per se or the degree of hypoxemia does not affect the outcome in critically ill patients with liver disease.^2^ It is the development of organ failures that contribute significantly to mortality. It was previously studied that patients with cirrhosis and three organ failures were unlikely to survive.^7^ However, it has been suggested to increase this threshold to four, due to improving survival.^6^ Since there is a call to shun prognostic pessimism in this group of patients,^6^ studying the weaning process would be apt. Both delayed and accelerated weaning from mechanical ventilation impose serious consequences on the patient outcome in terms of morbidity, mortality, and costs of ICU stay.^8^ Therefore, weaning should be done after accurate objective assessment and when the patient is truly ready for separation from the ventilator.

As a routine approach, the patient is assessed both subjectively by clinical assessment and objectively based on the gas exchange status (arterial blood oxygen saturation) and hemodynamic stability for the weaning process.^8^ Weaning failure can be defined as the failure of the spontaneous breathing trial (SBT) or the need for re-intubation within 48 hours of extubation.^1^

Additionally, the decision for extubation when based on the result of spontaneous breathing trial alone can have problems, as around between 10% and 20% of the patients experience respiratory insufficiency post-extubation and require re-intubation even after a successful SBT.^9^ Re-intubation further increases the morbidity and costs of ICU stay.^10^ Hence, this method should be combined with an accurate and objective assessment to minimize the probability of weaning failure. Thus, complications of re-intubation and delayed weaning can be avoided. Based on the studies conducted so far, the integrative weaning index (IWI) has a sensitivity of 97% and specificity of 94% in predicting the outcome of weaning from the mechanical ventilator.^11^ This index uses parameters that are easy to measure and independent of patient’s cooperation viz. the respiratory mechanics, the oxygenation, and the respiratory pattern, through the static compliance (Cst), arterial oxygen saturation (SaO_2_), and respiratory rate/tidal volume (f/Vt) ratio, respectively. Therefore in a single formula, it evaluates the respiratory mechanics, pattern, and oxygenation for the given patient. IWI is calculated by the following equation: IWI = Cst × SaO_2_/(f/V_T_), where Cst = static compliance of the respiratory system, SaO_2_ = arterial oxygen saturation, f = respiratory rate, and V_T_ = tidal volume.

## Methods

The present pilot study was conducted in the liver ICU with twenty beds, of a tertiary care center at New Delhi from October to December 2018 after approval from the Institutional Ethical Committee (F.25/5/107/ILBS/AC/2016/11252/2816-20). Adult cirrhotic patients who received mechanical ventilation for the first time in our institute for more than 24 hours were enrolled in the study. Patients who underwent a tracheostomy, outside intubations, re-intubation were excluded. The patients were enrolled in the study once the decision for weaning was taken by the ICU team.

The decision for weaning readiness and discontinuation of mechanical ventilation was made based on the following conditions: reversal of the cause/pathology of intubation, normothermia, no dyselectrolytemia, arterial blood gas analysis with pH > 7.3, SpO_2_ > 90% with a fraction of inspired oxygen (FiO_2_) ≤0.4 and a positive end-expiratory pressure (PEEP) <8 cm H_2_O, PaCO_2_ normal or baseline levels, hemodynamic stability (no or minimal dose of vasopressor drugs), and favorable level of consciousness (awake or easily arousable). After the decision for weaning was taken, the following parameters were noted: heart rate, blood pressure, respiratory rate, tidal volume, arterial oxygen saturation (SaO_2_) from arterial blood gas analysis, fraction of inspired oxygen (FiO_2_), positive end-expiratory pressure (PEEP), respiratory system compliance (Cst), total duration of mechanical ventilation, model for end-stage liver disease (MELD score), and sequential organ failure assessment score (SOFA score).

Weaning was initiated with the patients being placed on volume control mode for measurement of the plateau pressures measured by applying a 0.5-second inspiratory pause. Static compliance was further calculated by using the formula: tidal volume/(plateau pressure − PEEP). The patients were put on pressure support ventilation for further weaning, and the pressure support was gradually reduced to 6 cmH_2_O, PEEP, and FiO_2 _were maintained at 6 cm H_2_O and 40% respectively during weaning. The patients remained in these settings for 2 hours. The respiratory rate and tidal volume generated were recorded. Then SBT was given by using a T-piece with an oxygen flow of 10 L min^−1^ for 2 hours. Arterial blood gas analysis was done at the end of 2 hours of SBT. During the 2 hours of SBT, tolerance was continuously evaluated by the physician in charge. If the patient remained clinically stable after the 2 hours of SBT, the endotracheal tube was removed.

The extubated patient was then closely followed up for the next 48 hours. However, SBT was considered unsuccessful in case of any one of the following results: reduced SaO_2_ < 95%, PaO_2_ < 60 mm Hg, PaCO_2_ > 50 mm Hg, pH < 7.3, RR > 35/min, HR>140 min^−1^, systolic BP >180 and <90 mm Hg, agitation, perspiration, and reduced level of consciousness. In such a case, the patient was put back on the ventilator.

*IWI was calculated using the equation*:

IWI = Cst × SaO_2_/(f/V_T_), where Cst = static compliance of the respiratory system, SaO_2_ = arterial oxygen saturation, f = respiratory rate, VT = tidal volume, and f/V_T_ ratio = rapid shallow breathing index (RSBI). Then IWI was compared with the actual outcome of SBT.

### Statistical Analysis

The collected data was analyzed using the Statistical Package for Social Sciences version 22.0 software (IBM Corp.; Armonk, NY, USA). Data were expressed as mean ± standard deviation (SD) or median (interquartile range, IQR) as appropriate. For the analytical purpose, the study population was divided into two groups based on the outcome of SBT: successful SBT group (Group S) and unsuccessful SBT group (Group U). Categorical data and numerical data in both groups were compared using the chi-square test and student’s t-test, respectively. Sensitivity, specificity, positive predictive value, negative predictive value, diagnostic accuracy, and area under the receiver operating curve (AUC) were calculated for IWI. The Youden index was also calculated to find the most discriminative cut-off threshold value of IWI, to find out the appropriate one for this study population.

## Results

### Demographic Characteristics

About 236 patients were admitted to the ICU during the study period. Ninety-eight patients required mechanical ventilation for various indications. Thirty-six patients expired. Twelve patients underwent a tracheostomy. Four patients were reintubated. Eighteen patients were admitted with other diagnoses like acute liver failure, kidney disease, and pancreatitis. Three patients were excluded as they were aged <18 years. Thirty-eight patients did not meet the criteria for weaning due to their underlying critical illness. Twenty-seven patients met the inclusion criteria and were subsequently planned for SBT and weaning and were included for study analysis. Out of these, eighteen patients were successfully extubated (Group S) and nine patients failed the SBT (Group U).

The demographical data of the study population has been shown in Table 1. Both the groups were similar in their demographic variables of age, gender, and weight with no statistically significant differences (*P* > .05). Group S had more patients with encephalopathy as the primary reason for intubation; whereas, Group U had more patients with pneumonia as the primary reason for intubation. This difference was statistically significant. The rest of the factors were comparable in both groups. There was a significant difference between both the groups with respect to MELD scores (*P* < .001) and SOFA scores (*P* < .001). The mean MELD score in Group S was 20 (20–21) and that in Group U was 28 (27–29), and the mean SOFA score in Group S was 10 (9–10) whereas that in Group U was 13 (12–14).

Table 2 shows the clinical parameters studied in both groups during the process of weaning. The respiratory rate was significantly lower in Group S as compared to Group U (*P* < .001**)**. The tidal volume was significantly lower in Group U (*P* **= **.001). The arterial oxygen saturation level also showed a significant difference between the two groups (*P* = .019). The static compliance of the respiratory system and IWI were found to be significantly higher (*P* < .001) in Group S. However, there was no significant difference between the two groups with respect to heart rate, systolic and diastolic blood pressure. The patients in Group S were under mechanical ventilation for a significantly lesser duration than those in Group U [6 (5–7) vs 11 (7–13), 156; *P * = .005].

On subgroup analysis (Table 3) worsening pneumonia, hepatic encephalopathy, and upper gastrointestinal tract bleeding were the causes of intubation in sixteen, five, and six patients, respectively. Seven out of sixteen patients who were intubated for worsening pneumonia had successful SBT and the other nine patients had unsuccessful SBT (100% of this group). While, among those with hepatic encephalopathy and upper gastrointestinal tract bleeding, all the patients who were given spontaneous breathing trials (SBT) had a successful outcome.

**Figure 1.**
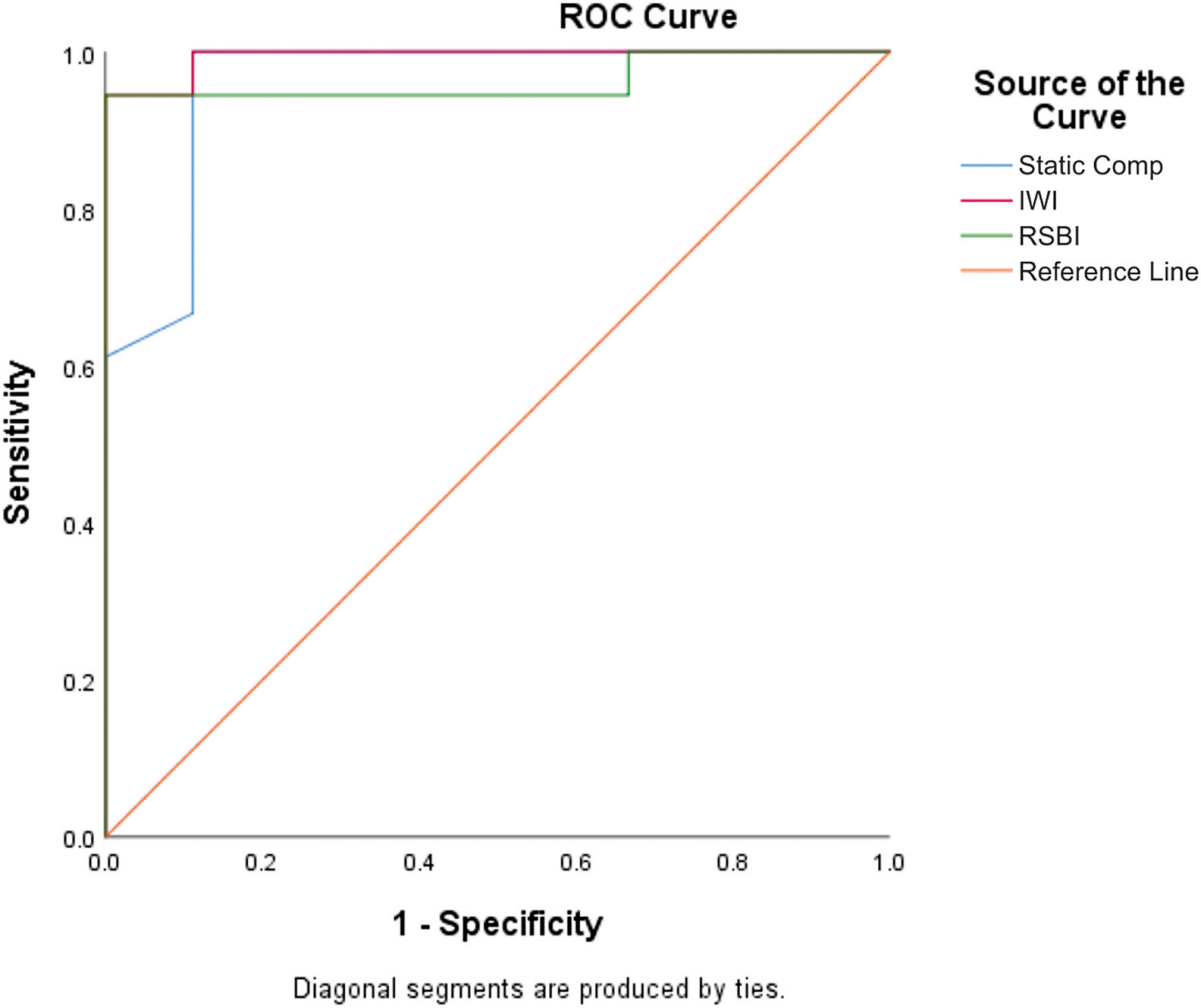
Showing receiver operating characteristics for variables associated with successful weaning. Static Comp, static compliance; IWI, integrative weaning index; RSBI, rapid shallow breathing index.

The static compliance, rapid shallow breathing index (RSBI), and IWI were highly significant predictors of weaning success (*P* < .001). The IWI had the highest area under the curve (AUC) of 99.4% followed by RSBI (96.3%) and static compliance (96%) (Figure 1). Youden index was 94.4 and 88.9 for threshold 28 mL/cmH_2_O breaths/min/L and 25 mL/cmH_2_O breaths/min/L, respectively (Table 4). The value of 25 mL/cmH_2_O breaths/min/L had a sensitivity of 88.9% and a specificity of 100%, higher negative predictive value (100%), and equivalent accuracy when compared to the threshold of 28 mL/cmH_2_O breaths/min/L where the corresponding values were 100% sensitivity and 94.4% specificity. The area under the receiver operating characteristic (AUC) curve was 99.4% for both the threshold values (Table 5).

## Discussion

This is the first study to evaluate weaning from mechanical ventilation in patients with cirrhosis. Studies for weaning from mechanical ventilation seldom distinguish the peculiarities of the critical patient. For example, studies for weaning do not individualize patients with chronic obstructive pulmonary disease (COPD) or acute respiratory distress syndrome (ARDS).^12^ These patients show changes of chronic lung disease and intense inflammatory response, respectively. Likewise, patients with cirrhosis represent a unique subset of patients for the weaning process. Risk factors like encephalopathy, gastrointestinal bleed, alcohol withdrawal, nutritional deficiencies influence the process of weaning, apart from pulmonary issues.

Recent studies have shown a decline in mortality in patients with liver disease and multi-organ failure admitted to the ICU.^6^ These improvements most likely reflect a general trend of attention to detail to critical care systems management (fluids, infection control, ventilation techniques, renal and cardiovascular parameters, and nutrition). In our study too, the patients in Group S were successfully weaned despite advanced liver failure (MELD 20) and a high SOFA score of 10.

All our patients in Group U were intubated for pneumonia. It has been found that even in the early stages of liver disease, there is inhibition of the signal transducer and activator of transcription 3 (Stat3) pathway of hepatocytes.^13^ Inhibition of this hepatocyte STAT 3 response is associated with higher lung and blood bacteria burden. In addition, immunological abnormalities hinder the control of lung pathogens.^14^ This could result in prolonged treatment and could be the cause of prolonged ventilation in our patients in Group U. Prolonged mechanical ventilation is known to cause diaphragmatic protein break down and reduced protein synthesis.^15^ This causes diaphragm dysfunction and is significantly associated with weaning failure.^16^ Patients in Group U also had a significantly higher respiratory rate and low respiratory compliance compared to Group S. Hyperventilation is common in liver disease. The exact cause is not known.^17^ Hyperventilation causes excessive use of the respiratory muscles, which may be mildly impaired. This increased respiratory drive coupled with a muscle weakness causes a neuromechanical dissociation and leads to a feeling of dyspnea in this group of patients.^17^ Neuromuscular weakness coupled with diaphragm weakness could also have contributed to the low respiratory compliance in Group U. Compliance contributes to 65% of the total work of breathing.^18^ Therefore, lower compliance increases the work of breathing and can lead to weaning failure.

Respiratory compliance, RSBI, and IWI were found to be significant predictors of weaning success in our study. Nemer et al.^11^ found that respiratory compliance of >30 mL/cmH_2_O and an RSBI of <100 breaths/min/L were the significant predictors of weaning success. We found that a respiratory compliance of >34 mL/cmH_2_O and an RSBI of <75 breaths/min/L were predictors of weaning success in patients with liver disease. Their patient cohort mainly consisted of patients with COPD, pneumonia, and postoperative acute respiratory failure. They found an IWI of 25 mL/cmH_2_O breaths/min/L to differentiate between successful and unsuccessful weaning. It had a sensitivity of 97% and a specificity of 94% to predict successful weaning. The robustness of this index has been established due to its ability to evaluate the respiratory mechanics, oxygenation, and respiratory pattern in a single equation. It has been proven to be reliable in subsequent studies by El-Baradey et al.^19^ and Ebrahimabadi et al.^20^ Multiplying the compliance by the saturation in the numerator detect the ability to maintain oxygenation, with good or bad respiratory mechanics. Dividing this by the f/VTratio (RSBI) in the denominator detects those who will or will not be able to maintain unassisted breathing. Cst and SaO_2 _are inversely proportional to the f/Vt ratio (RSBI). The higher the Cst and SaO_2_, the lower is the f/Vt ratio and IWI tends to be higher.^11^

We found that a cut-off of 25 mL/cmH_2_O breaths/min/L had a sensitivity of 88.9% and a specificity of 100%, whereas a higher cut-off of 28 mL/cmH_2_O breaths/min/L had a sensitivity of 100% and a specificity of 94%. In the context of mechanical ventilator weaning, the sensitivity of a measure is defined as its ability to accurately identify, from among the patients, who present with a positive index (e.g., IWI ≥ 28 mL/cmH_2_O breaths/min/L), the proportion of those in whom weaning will be successful.^21^ Conversely, the specificity of a measure is defined as its ability to identify, from among the patients, who present with a negative index (e.g., IWI < 28 mL/cmH_2_O breaths/min/L), the proportion of those in whom weaning will fail. Since the value of 28 mL/cmH_2_O breaths/min/L has 100% sensitivity and a reasonable good specificity, we suggest the higher value to be a better predictor of weaning.

The robustness of the higher value could be explained by the inherent increased central respiratory drive seen in cirrhosis. This increases the work of breathing and oxygen demand. Hence they require higher tidal volumes and compliance to maintain the adequacy of oxygenation. Therefore, those who can match the respiratory drive with a higher tidal volume and compliance are successfully weaned and may fare better after extubation.

Among the patients who were extubated, none had extubation failure with an IWI of more than 25 mL/cmH_2_O breaths/min/L. However, this study has deduced that a threshold of 28 mL/cmH_2_O breaths/min/L is a better predictor of successful SBT based on the Youden index. This threshold of IWI can contribute to improving weaning success in cirrhotic patients.

The limitations of this study are as follows:

1. Being a pilot study, the population is small.

2. Measurement of static compliance is technically challenging since it requires identifying ventilator breath and applying inspiratory hold in VCV mode in a spontaneously breathing patient.

## Conclusion

IWI is a successful predictor for weaning success among cirrhotic patients. A threshold value of 28 mL/cmH_2_O breaths/min/L is useful to identify the patients suitable for weaning and extubation. However, it requires further study in a larger population.

## Figures and Tables

**Table 1.** Demographic Parameters of Study Population

	Group S (n = 18), Median (IQR), Mean ± SD	Group U (n = 9), Median (IQR), Mean ± SD	*P*
Age (years)	55 (40–58), 53.61 ± 9.81	57 (46–60), 58.33 ± 3.53	.357
Gender (M/F)	14/4	5/4	.38
Weight (kg)	60 (55–70), 62.22 ± 8.94	60 (55–60), 58.33 ± 3.53	.120
MELD score	20 (20–21), 20.50 ± 1.24	28 (27–29), 27.56 ± 3.08	<.001
SOFA score	10 (9–10), 9.50 ± 0.78	13 (12–14), 12.56 ± 1.23	<.001
Comorbidities	N (%)	N (%)	
Hypertension	7/18 ( 38%)	4/9 (44%)	.78
Diabetes mellitus	8/18 (44%)	3/9 (33%)	.58
Hypothyroidism	4/18 (22%)	3/9 (33%)	.65
Modifiable factors	N (%)	N (%)	
Tense ascites	10/18 (55%)	6/9 (66%)	.58
Hydrothorax	4/18 (22%)	2/18 (22%)	1.00
Encephalopathy	5/18 (27%)	0/9 (0)	.01
Pneumonia	7/18 (38%)	9/9 (100%)	0.01

**Table 2.** Observed Variables in the Study Population

Parameters	Outcome of SBT				*P*
Group S (n = 18)		Group U (n = 9)	
	Mean ± SD	Median (IQR)	Mean ± SD	Median (IQR)	
HR (bpm)	81.22 ± 6.17	81 (77–86)	82 ± 5.47	81 (77–87)	.743
SBP (mm Hg)	125 ± 8.34	126 (118–132)	126.33 ± 6.46	128 (122–130)	.652
DBP (mm Hg)	60.83 ± 6.36	60 (55–71)	64.44 ± 6.61	60 (55–60)	.182
RR (bpm)	19.44 ± 2.79	19 (18–22)	26.11 ± 2.14	26 (24–28)	<.001
TV (L/breath)	0.347 ± 0.072	0.377 (0.292–0.390)	0.265 ± 0.039	0.260 (0.240–0.282)	.001
Static compliance (mL/cmH_2_O)	45.96 ± 10.53	42.45 (37.35–52.20)	27.15 ± 5.51	28.40 (22.50–29.70)	<.001
IWI (mL/cmH_2_O breaths/min/L)	80.39 ± 38.60	71.16 (57.34–99.62)	22.99 ± 2.34	22.83 (21.33–24.63)	<.001
SaO_2 _(%)	97.81 ± 0.93	98.1 (97.22–98.47)	96.85 ± 0.93	96.7 (96.40–97.45)	**.019**
Mechanical ventilation duration in days	6.72 ± 2.76	6 (5–7)	10.44 ± 3.35	11 (7–13)	**.005**

SBT, spontaneous breathing trial; SD, standard deviation; IQR, interquartile range; HR, heart rate in beats per minute (bpm); SBP, systolic blood pressure; DBP, diastolic blood pressure; RR, respiratory rate in breaths per minute (bpm); TV, tidal volume; IWI, inspiratory weaning index; SaO_2_, arterial oxygen saturation.

**Table 3.** Outcome of SBT According to Cause of Intubation

Cause of Intubation	Group S	Group U
Pneumonia	7 (28%)	9 (100%)
Hepatic encephalopathy	5 (100%)	0
Upper GI bleed	6 (100%)	0

SBT, spontaneous breathing trial.

**Table 4.** Comparing the Accuracy of IWI According to Different Threshold Values

Threshold (mL/cmH_2_O breaths/min/L)	Sensitivity (%)	Specificity (%)	PPV (%)	NPV (%)	Accuracy (%)	Youden Index
28	100	94.4	100	90	96.3	94.4
25	88.9	100	94.7	100	96.3	88.9

IWI, integrative weaning index; PPV, positive predictive value; NPV, negative predictive value.

**Table 5.** AUC for Different Threshold of IWI

Threshold	AUC	Standard Error	*P*
28	99.4	0.01	<.001
25	99.4	0.06	<.001

AUC, area under the curve; IWI, integrative weaning index.
